# Tazemetostat for relapsed/refractory B-cell non-Hodgkin lymphoma with EZH2 mutation in Japan: 3-year follow-up for a phase II study

**DOI:** 10.1007/s12185-024-03834-9

**Published:** 2024-08-23

**Authors:** Koji Izutsu, Kiyoshi Ando, Momoko Nishikori, Hirohiko Shibayama, Hideki Goto, Junya Kuroda, Koji Kato, Yoshitaka Imaizumi, Kisato Nosaka, Rika Sakai, Maho Abe, Seiichiro Hojo, Tadashi Nakanishi, Shinya Rai

**Affiliations:** 1 Department of Hematology, National Cancer Center Hospital, 5-1-1 Tsukiji, Chuo-ku, Tokyo, 104-0045 Japan; 2 Department of Hematology and Oncology, Tokai University School of Medicine, Isehara, Japan; 3 Department of Hematology and Oncology, Kyoto University, Kyoto, Japan; 4 Department of Hematology and Oncology, Osaka University Hospital, Suita, Japan; 5 Department of Hematology, Hokkaido University Hospital, Sapporo, Japan; 6 Division of Hematology and Oncology, Kyoto Prefectural University of Medicine, Kyoto, Japan; 7 Department of Medicine and Biosystemic Science, Kyushu University Graduate School of Medical Sciences, Fukuoka, Japan; 8 Department of Hematology, Nagasaki University Hospital, Nagasaki, Japan; 9 Department of Hematology, Rheumatology, and Infectious Diseases, Kumamoto University, Kumamoto, Japan; 10 Department of Hematology and Medical Oncology, Kanagawa Cancer Center, Yokohama, Japan; 11 Eisai Co., Ltd., Tokyo, Japan; 12 Department of Hematology and Rheumatology, Faculty of Medicine, Kindai University, Osakasayama, Japan

**Keywords:** *EZH2* mutation, B-cell non-Hodgkin lymphoma, Phase II, Tazemetostat, Trimethylation of lysine 27 in histone 3

## Abstract

Previously, we reported the efficacy and safety of tazemetostat in Japanese patients with relapsed/refractory follicular lymphoma (FL) and diffuse large B-cell lymphoma (DLBCL) harboring the *EZH2* mutation in a multicenter, open-label, phase II study. Here, we present a follow-up analysis of tazemetostat at a long-term median follow-up of 35.0 months. Twenty patients were enrolled: 17 in the FL cohort and three in the DLBCL cohort. In the FL cohort, the objective response rate was 70.6%, consistent with the primary analysis, and the median progression-free survival (PFS) was not reached. The 24-month and 36-month PFS rates were 72.1% (95% confidence interval [CI] 41.5%–88.6%) and 64.1% (95% CI 33.7%–83.4%), respectively. The median duration of treatment was 30.2 months. After the primary analysis at a median follow-up of 12.9 months, grade 1–2 urinary tract infection, peripheral motor neuropathy, and hypogammaglobulinemia newly emerged, but the incidence of adverse events (AEs) did not increase notably during this follow-up period. No unexpected grade ≥ 3 treatment-related AEs were reported. Long-term oral monotherapy with tazemetostat showed favorable efficacy and safety profiles, indicating that it may be a useful third-line or later treatment option for patients with relapsed/refractory FL harboring the *EZH2* mutation. *Trial registration:* ClinicalTrials.gov: NCT03456726.

## Introduction

Tazemetostat is a first-in-class, selective, reversible, small-molecule, oral inhibitor of enhancer of zeste homolog 2 (EZH2), a histone methyltransferase enzyme responsible for the methylation of histone H3K27 that plays a role in the epigenetic regulation of various genes [1, 2]. Although the detailed mechanism of action has not been elucidated, tazemetostat is thought to inhibit tumor growth by inhibiting the methylation of H3K27 by mutant and wild-type *EZH2*, which results in cell cycle arrest and apoptosis induction.

Two phase II studies of tazemetostat monotherapy for B-cell non-Hodgkin lymphoma (B-NHL) were conducted in multiple countries, including Japan, to investigate the efficacy and safety of tazemetostat. In the global phase II study (NCT01897571), patients with relapsed/refractory follicular lymphoma (FL) achieved an objective response rate (ORR) of 69% (95% confidence interval [CI] 53%–82%; 31 of 45 patients) in the mutant *EZH2* cohort and 35% (95% CI 23%–49%; 19 of 54 patients) in the wild-type *EZH2* cohort (median follow-up period of 22.0 months) [3]. Based on these results, tazemetostat monotherapy received accelerated approval in the US for the treatment of relapsed/refractory FL in 2020 [3]. We conducted another phase II study (NCT03456726) for Japanese patients with relapsed/refractory FL harboring the *EZH2* mutation who demonstrated a consistent ORR of 76.5% (90% CI 53.9%–91.5%; 13 of 17 patients) during a median follow-up period of 12.9 months [4]. Based on the results of both studies, tazemetostat monotherapy received regulatory approval in Japan for *EZH2* mutation-positive relapsed/refractory FL in 2021.

However, because of the limited follow-up periods of reported studies, the long-term efficacy and safety of tazemetostat remains unclear. For example, at the data cutoff of the phase II study in Japan, treatment was ongoing in 76.5% and 66.7% of patients in the FL cohort and the diffuse large B-cell lymphoma (DLBCL) cohort, respectively. Considering the generally favorable prognosis of patients with FL, it is essential to understand the long-term efficacy and safety profile. Therefore, we conducted an ad hoc follow-up analysis of the Japanese phase II study to investigate the long-term efficacy and safety of tazemetostat.

## Methods

### Study design

This study was an open-label, single-arm, phase II Japanese study of tazemetostat, and we previously reported the results [4]. Herein, we report the follow-up results.

### Ethics

The study adhered to the principles of the World Medical Association Declaration of Helsinki, Good Clinical Practice guidelines, and local regulations. Patients provided written informed consent for tumor *EZH2* mutation testing and clinical study participation. The institutional review board of each site approved the study protocol.

### Patients

Detailed eligibility criteria have been previously reported [4]. The key inclusion criteria were a histological diagnosis of FL or DLBCL (including primary mediastinal large B-cell lymphoma and transformed FL); *EZH2* mutation of the tumor confirmed by the central laboratory using the cobas^®^ EZH2 Mutation Test (Roche Molecular Systems, Inc.); previous systemic chemotherapy or antibody therapy; disease progression, non-response or relapse/progression after the last treatment without standard treatment options; and no carry-over of Grade ≥ 2 adverse events (AEs) from the prior treatment that may have affected the safety evaluation of tazemetostat.

### Treatment

Tazemetostat monotherapy (800 mg twice daily) was administered orally in 28-day continuous cycles until disease progression (except when the investigator decided treatment should continue), development of unacceptable toxicity, or patient request to discontinue. The dosage of tazemetostat in the phase II study was determined based on the safety and efficacy reported in previous studies [3, 5].

The criteria for treatment interruption were as follows: intolerable Grade ≥ 2 toxicity or neutropenia with an absolute neutrophil count of ≤ 0.75 × 10^9^/L, except for Grade 3 thrombocytopenia or anemia, Grade 3 or 4 leukopenia, and laboratory abnormalities that were not clinically relevant. Once these parameters were recovered, the treatment could be reinstated at a reduced dose based on the previous dose level of 600 mg and 400 mg twice daily.

### Outcomes and assessments

The efficacy endpoints of the study were evaluated according to the revised response criteria for malignant lymphoma (IWG-2007) [6]. The primary endpoint was ORR (i.e., rate of complete response [CR] or partial response [PR] assessed as the individual patient’s best overall response [BOR]). Secondary endpoints were PFS, defined as the time from the first dose of study treatment to established progressive disease (PD) or death from any cause; duration of response (DOR), defined as the time from initial confirmation of response to the first documented evidence of PD; time to response (TTR), defined as the time from the first dose to the time of the first response; and safety.

Tumor evaluations were conducted every 8 weeks up to 32 weeks, every 12 weeks thereafter, and at discontinuation. The independent radiological review committee and investigators evaluated the tumor assessment results.

The safety endpoints were the incidence of treatment-emergent AEs (TEAEs) and treatment-related AEs (TRAEs), as determined by the investigator. AEs were coded using the Medical Dictionary for Regulatory Activities version 22.0. Classification of AE severity was based on the Common Terminology Criteria for Adverse Events (version 4.03). For the analysis of TRAE onset, the occurrence of a TRAE was categorized by the specific period of onset: < 6 months, 6–12 months, and > 12 months.

### Statistical analysis

Time‐to‐event endpoints were estimated using the Kaplan–Meier method. SAS for Windows (version 9.2 or later, SAS Institute Inc., Cary, NC) was used for statistical analysis.

## Results

### Patients

This study was initiated in April 2018 at 28 sites in Japan and completed in December 2021 as all patients had discontinued the study, with a median follow-up period of 35.0 months. In total, 20 patients were enrolled in the study, 17 of whom had FL and three had DLBCL. Based on the sponsor’s decision not to pursue the development of tazemetostat for DLBCL, new enrollment for the DLBCL cohort was stopped partway after the study started. Details on the patient population and enrollment have been reported previously [4].

Table 1 summarizes the baseline characteristics of patients. In the FL cohort, patients had a median (range) age of 66.0 (46–81) years, 52.9% were female, and 94.1% had an Eastern Cooperative Oncology Group performance status of 0. No patients in either cohort had B symptoms, 17.6% had bulky lesions, and 23.5% met the GELF criteria. The proportion of patients who were refractory to the last treatment regimen was 17.6%. Four patients (23.5%) had elevated lactate dehydrogenase.

**Table 1:** Patient characteristics

	FL (*n* = 17)	DLBCL (*n* = 3)	Total (*n* = 20)
Age, years, median (range)^a^	66.0 (46–81)	71.0 (70–83)	69.5 (46–83)
Age ≥ 65 years, *n* (%)	9 (52.9)	3 (100.0)	12 (60.0)
Female, *n* (%)	9 (52.9)	2 (66.7)	11 (55.0)
ECOG PS, *n* (%)			
0	16 (94.1)	3 (100.0)	19 (95.0)
1	1 (5.9)	0	1 (5.0)
B symptoms, *n* (%)	0	0	0
Bulky lesions, *n* (%)^b^	3 (17.6)	1 (33.3)	4 (20.0)
Satisfied GELF criteria, *n* (%)^c^	4 (23.5)	NA	NA
Ann Arbor staging, *n* (%)			
Stage I	3 (17.6)	0	3 (15.0)
Stage II	2 (11.8)	3 (100.0)	5 (25.0)
Stage III	6 (35.3)	0	6 (30.0)
Stage IV	6 (35.3)	0	6 (30.0)
Baseline bone marrow assessment, *n* (%)			
Positive	5 (29.4)	0	5 (25.0)
Negative	12 (70.6)	3 (100.0)	15 (75.0)
Median time from initial diagnosis, years^d^	6.0	15.7	7.0
Previous systemic chemotherapy regimens, median (range)	2.0 (1–4)	4.0 (1–5)	2.0 (1–5)
Refractory to the last treatment regimen, *n* (%)^e,f^			
Yes	3 (17.6)	0	3 (15.0)
No	12 (70.6)	2 (66.7)	14 (70.0)
LDH elevated	4 (23.5)	1 (33.3)	5 (25.0)

*DLBCL* Diffuse large B-cell lymphoma, *ECOG* Eastern Cooperative Oncology Group, *FL* follicular lymphoma, *GELF* Groupe d’Etude des Lymphomes Folliculaires, *NA* not available, *LDH* lactate dehydrogenase, *PS* performance status

^a^Age was calculated at the date of informed consent

^b^Bulky lesions were defined as a target lesion of > 7 cm in diameter or three nodal target lesions of > 3 cm in diameter each

^c^Defined as a target lesion of > 7 cm in diameter, at least three nodal target lesions of > 3 cm in diameter each, the presence of B symptoms at baseline, a concentration of serum LDH higher than the upper limit of normal, a serum hemoglobin concentration of ≤ 100 g/L, a neutrophil count of ≤ 1500 cells/μL, or a platelet count of ≤ 100,000 platelets/mL

^d^If the year and month were not missing, but the day was missing, the missing day was set to the 15th. If the year was not missing, but the month was missing, the month and day were set to July 1st

^e^Subjects who had previously received systemic chemotherapy

^f^Denominator includes patients with response to the last regimen of complete response, partial response, stable disease, or progressive disease; not evaluable/not applicable/unknown/missing were excluded

The median (range) number of dosing cycles was 33.0 (3–45) cycles. The median (range) duration of treatment was 30.2 (2.8–40.5) months, and the median (range) relative dose intensity was 97.8% (56.0%–99.8%). The reasons for discontinuation were disease progression (6 [35.3%] and 1 [33.3%] in the FL and DLBCL cohorts, respectively), AEs (4 [23.5%] and 2 [66.7%]), patient request (1 [5.9%] in the FL cohort) and a switch from the study drug to commercial tazemetostat (TAZVERIK^®^; 6 [35.3%] in the FL cohort).

### Efficacy by investigator assessment

#### Primary endpoint: BOR

**Fig. 1:**
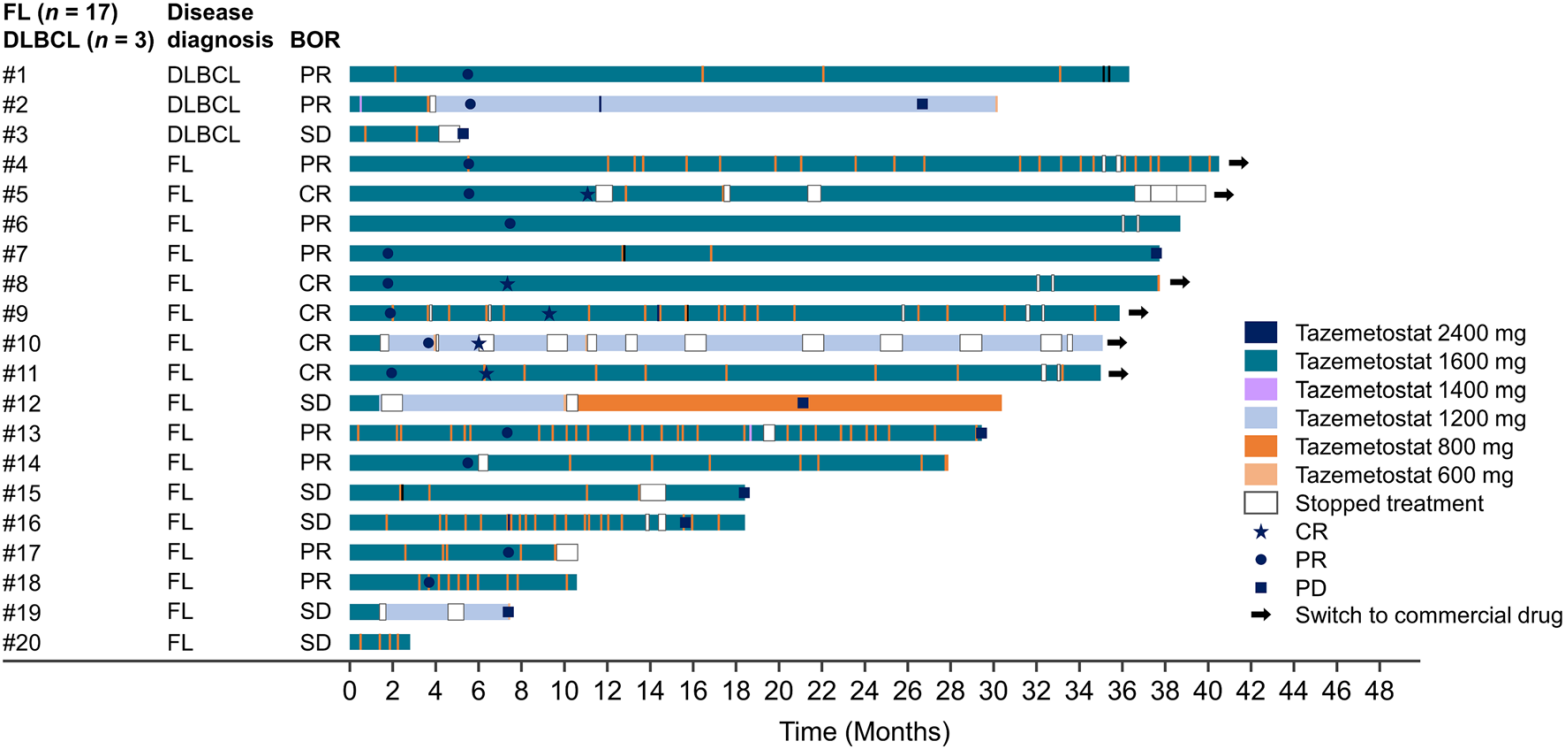
Duration of treatment and overall timepoint assessments by investigator assessment. *BOR* best overall response, *FL* follicular lymphoma, *DLBCL* diffuse large B-cell lymphoma, *PR* partial response, *SD* stable disease, *CR* complete response, *PD* progressive disease

Each patient’s BOR based on investigator assessment is shown in the swimmer plot (Fig. 1). Among the 17 patients in the FL cohort, the ORR was 70.6% (90% CI 47.8%–87.6%) by investigator assessment. Compared with the primary analysis [4], there was no change in BOR. Five patients (29.4%) achieved CR; seven (41.2%), PR; and five (29.4%), stable disease (SD). No patients had PD. Among the 11 patients (64.7%) with BOR who continued treatment for > 2 years, CR was achieved in five, PR in five, and SD in one. Five patients (29.4%) continued treatment for > 3 years, with a BOR of CR in two and PR in three. Six patients (35.3%) discontinued treatment within 2 years: two had PR, four had SD, and none had CR.

One patient (Patient #10 in Fig. 1) who achieved CR continued treatment for 35.0 months with a dose reduction to 600 mg twice daily and 12 drug interruptions in total. The reasons for this patient’s drug interruptions were dysgeusia (seven times), colds (twice), and hyperkalemia, influenza, and coronavirus vaccination (once each).

#### Secondary endpoints: PFS, DOR, and TTR

**Fig. 2:**
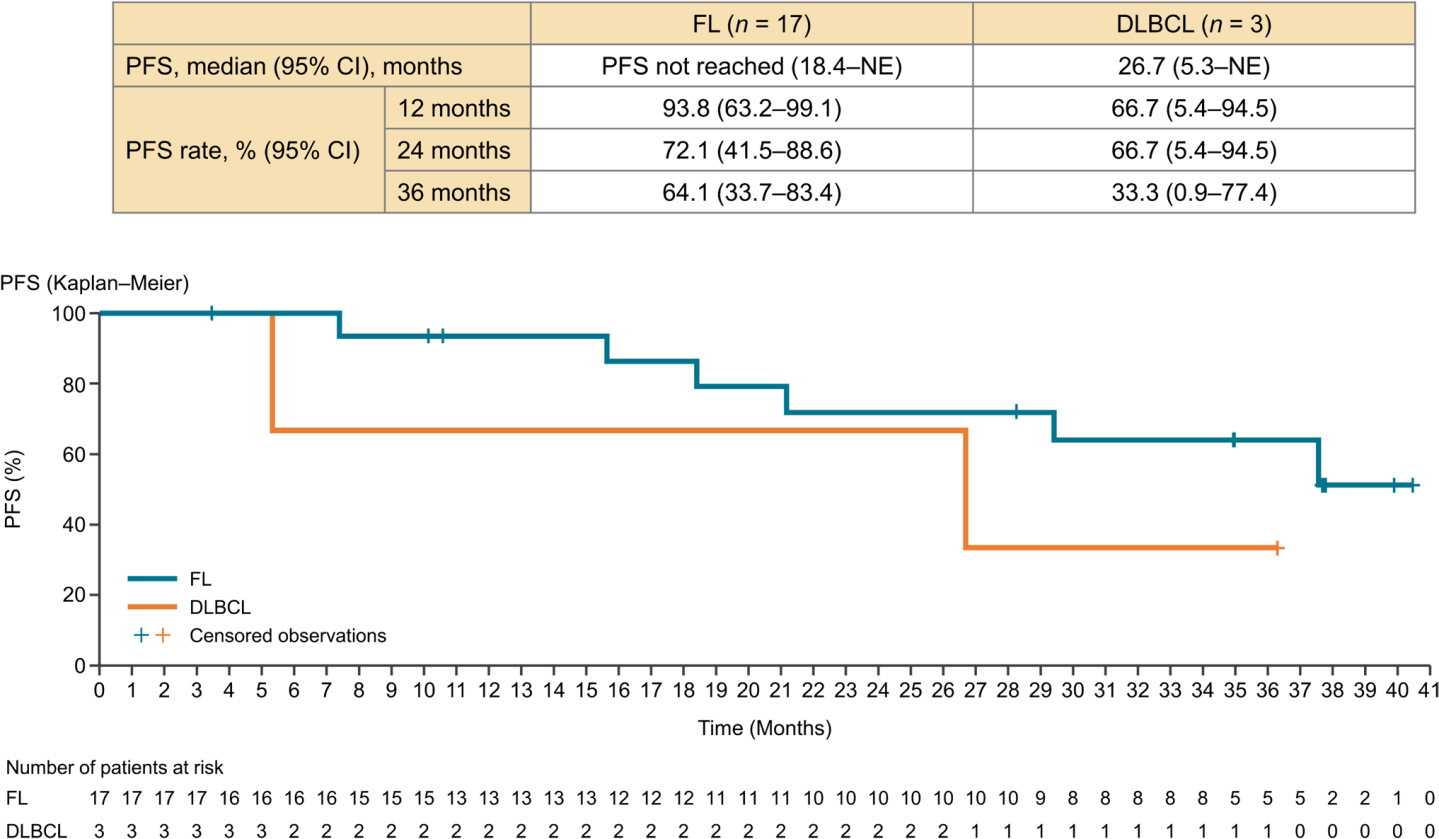
Progression-free survival by investigator assessment. *FL* follicular lymphoma, *DLBCL* diffuse large B-cell lymphoma, *PFS* progression-free survival, *CI* confidence interval, *NE* not evaluable

**Fig. 3:**
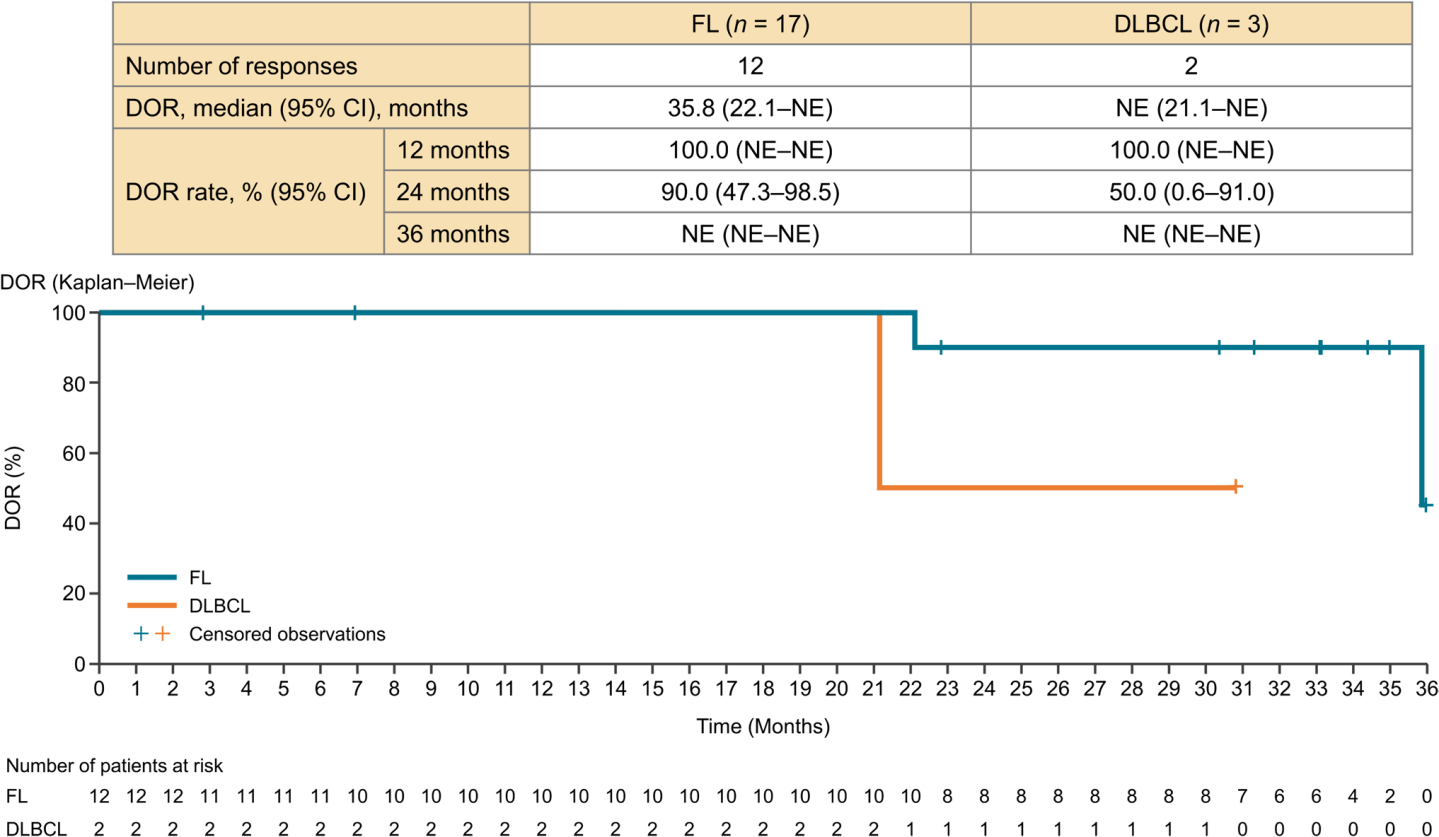
Duration of response by investigator assessment. *DOR* duration of response, *FL* follicular lymphoma, *DLBCL* diffuse large B-cell lymphoma, *CI* confidence interval, *NE* not evaluable

**Fig. 4:**
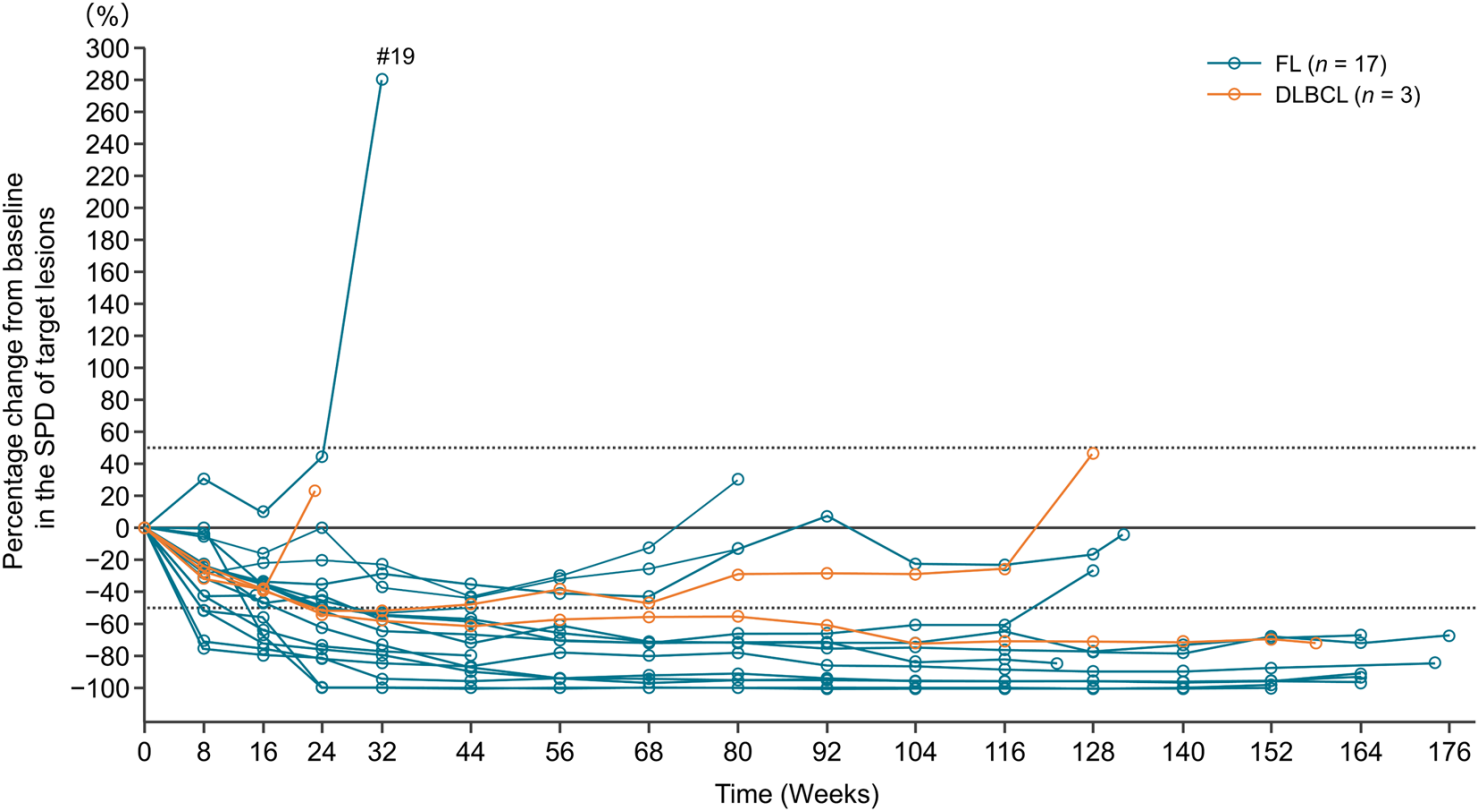
Percentage change from baseline in the SPD of target lesions by investigator assessment. *FL* follicular lymphoma, *DLBCL* diffuse large B-cell lymphoma, *SPD* sum of the products of the perpendicular diameters

In the FL cohort, the median PFS was not reached (95% CI 18.4 months–not estimable). The 24-month PFS rate was 72.1% (95% CI 41.5%–88.6%), and the 36-month median PFS rate was 64.1% (95% CI 33.7%–83.4%) (Fig. 2). In the FL cohort, the median DOR was 35.8 months (95% CI 22.1–not estimable), and the 24-month DOR rate was 90.0% (95% CI 47.3%–98.5%) (Fig. 3). The percentage change from baseline in the target lesion per investigator assessment is shown in a spider plot (Fig. 4). Of the 12 patients who achieved a response, five (41.7%) maintained a response for at least 3 years, and 10 (83.3%) maintained a response for at least 2 years. In one case of FL (Patient #19), there was an increase in the SPD observed early during the treatment period, at approximately 6 months. The patient was initially diagnosed with Stage I FL, with a target lesion located in the para-aortic region (SPD: 1550.4 mm^2^). The lesion, with an average tumor burden in this study, was refractory to the most recent chemotherapy treatment. The patient experienced treatment interruption due to Grade 3 neutropenia and Grade 3 mechanical ileus. Ultimately, treatment was discontinued on day 225 because of disease progression (SPD: 5899.9 mm^2^). In the FL cohort, the median (range) TTR was 4.59 (1.8–7.5) months.

### Safety

In the FL cohort, tazemetostat was interrupted in 11 patients (64.7%); three patients (17.6%) experienced dose reductions, with a median time to first dose reduction of 1.9 months.

Table 2 summarizes the safety results observed in the primary analysis (from December 2019) and the follow-up analysis (from December 2021). In the follow-up analysis among a total of 20 patients, TEAEs were reported in 20 patients (100.0%), and Grade ≥ 3 TEAEs occurred in 11 patients (55.0%). The most frequent TEAEs were dysgeusia in 10 patients (50.0%), nasopharyngitis in eight patients (40.0%), and lymphopenia, constipation, stomatitis, upper respiratory tract infection, and blood creatine phosphokinase increased in five patients (25.0%) each.

**Table 2:** Treatment-emergent adverse events (TEAEs) occurring in two or more patients and treatment-related AEs (TRAEs)

Number of patients (%)	TEAEs				TRAEs			
	Primary analysis: as of December 2019 [4] (*N* = 20)		Follow-up analysis: as of December 2021 (*N* = 20)		Primary analysis: as of December 2019 [4] (*N* = 20)		Follow-up analysis: as of December 2021 (*N* = 20)	
	Any grade	Grade ≥ 3	Any grade	Grade ≥ 3	Any grade	Grade ≥ 3	Any grade	Grade ≥ 3
Any	20 (100.0)	8 (40.0)	20 (100.0)	11 (55.0)	20 (100.0)	6 (30.0)	20 (100.0)	6 (30.0)
Dysgeusia	10 (50.0)	0 (0.0)	10 (50.0)	0 (0.0)	10 (50.0)	0 (0.0)	10 (50.0)	0 (0.0)
Nasopharyngitis	7 (35.0)	0 (0.0)	8 (40.0)	0 (0.0)	2 (10.0)	0 (0.0)	2 (10.0)	0 (0.0)
Lymphopenia	5 (25.0)	2 (10.0)	5 (25.0)	3 (15.0)	5 (25.0)	2 (10.0)	5 (25.0)	3 (15.0)
Blood creatine phosphokinase increased	5 (25.0)	0 (0.0)	5 (25.0)	0 (0.0)	1 (5.0)	0 (0.0)	1 (5.0)	0 (0.0)
Constipation	4 (20.0)	0 (0.0)	5 (25.0)	0 (0.0)	2 (10.0)	0 (0.0)	2 (10.0)	0 (0.0)
Upper respiratory tract infection	4 (20.0)	0 (0.0)	5 (25.0)	0 (0.0)	1 (5.0)	0 (0.0)	1 (5.0)	0 (0.0)
Neutropenia	3 (15.0)	1 (5.0)	3 (15.0)	1 (5.0)	3 (15.0)	1 (5.0)	3 (15.0)	1 (5.0)
Thrombocytopenia	3 (15.0)	0 (0.0)	3 (15.0)	0 (0.0)	3 (15.0)	0 (0.0)	3 (15.0)	0 (0.0)
Nausea	3 (15.0)	0 (0.0)	3 (15.0)	0 (0.0)	2 (10.0)	0 (0.0)	2 (10.0)	0 (0.0)
Stomatitis	3 (15.0)	0 (0.0)	5 (25.0)	0 (0.0)	3 (15.0)	0 (0.0)	3 (15.0)	0 (0.0)
Weight decreased	3 (15.0)	0 (0.0)	3 (15.0)	0 (0.0)	2 (10.0)	0 (0.0)	2 (10.0)	0 (0.0)
Alopecia	3 (15.0)	0 (0.0)	3 (15.0)	0 (0.0)	3 (15.0)	0 (0.0)	3 (15.0)	0 (0.0)
Rash	3 (15.0)	0 (0.0)	4 (20.0)	0 (0.0)	1 (5.0)	0 (0.0)	1 (5.0)	0 (0.0)
Anemia	2 (10.0)	0 (0.0)	2 (10.0)	0 (0.0)	2 (10.0)	0 (0.0)	2 (10.0)	0 (0.0)
Fatigue	2 (10.0)	0 (0.0)	2 (10.0)	0 (0.0)	1 (5.0)	0 (0.0)	1 (5.0)	0 (0.0)
Malaise	2 (10.0)	0 (0.0)	2 (10.0)	0 (0.0)	2 (10.0)	0 (0.0)	2 (10.0)	0 (0.0)
Herpes simplex	2 (10.0)	0 (0.0)	2 (10.0)	0 (0.0)	1 (5.0)	0 (0.0)	1 (5.0)	0 (0.0)
Pneumonia	2 (10.0)	1 (5.0)	2 (10.0)	1 (5.0)	1 (5.0)	1 (5.0)	1 (5.0)	1 (5.0)
Alanine aminotransferase increased	2 (10.0)	0 (0.0)	1 (5.0)	0 (0.0)	2 (10.0)	0 (0.0)	1 (5.0)	0 (0.0)
Amylase increased	2 (10.0)	0 (0.0)	2 (10.0)	0 (0.0)	2 (10.0)	0 (0.0)	2 (10.0)	0 (0.0)
Aspartate aminotransferase increased	2 (10.0)	0 (0.0)	1 (5.0)	0 (0.0)	2 (10.0)	0 (0.0)	1 (5.0)	0 (0.0)
Blood creatinine increased	2 (10.0)	0 (0.0)	3 (15.0)	0 (0.0)	2 (10.0)	0 (0.0)	2 (10.0)	0 (0.0)
Electrocardiogram QT prolonged	2 (10.0)	0 (0.0)	2 (10.0)	0 (0.0)	2 (10.0)	0 (0.0)	2 (10.0)	0 (0.0)
Hypophosphatemia	2 (10.0)	0 (0.0)	2 (10.0)	0 (0.0)	2 (10.0)	0 (0.0)	2 (10.0)	0 (0.0)
Eczema	2 (10.0)	0 (0.0)	2 (10.0)	0 (0.0)	2 (10.0)	0 (0.0)	2 (10.0)	0 (0.0)
Conjunctival hemorrhage	1 (5.0)	0 (0.0)	2 (10.0)	0 (0.0)	0 (0.0)	0 (0.0)	0 (0.0)	0 (0.0)
Pyrexia	1 (5.0)	0 (0.0)	2 (10.0)	1 (5.0)	0 (0.0)	0 (0.0)	0 (0.0)	0 (0.0)
Influenza	2 (10.0)	0 (0.0)	2 (10.0)	0 (0.0)	0 (0.0)	0 (0.0)	0 (0.0)	0 (0.0)
Periodontitis	1 (5.0)	0 (0.0)	2 (10.0)	1 (5.0)	0 (0.0)	0 (0.0)	0 (0.0)	0 (0.0)
Urinary tract infection	1 (5.0)	0 (0.0)	2 (10.0)	0 (0.0)	0 (0.0)	0 (0.0)	1 (5.0)	0 (0.0)
Hypertriglyceridemia	2 (10.0)	1 (5.0)	2 (10.0)	1 (5.0)	0 (0.0)	0 (0.0)	0 (0.0)	0 (0.0)

MedDRA version 22.0; CTCAE version 4.03

Of the ten patients who developed dysgeusia (Grade 1: nine patients, Grade 2: one patient), seven continued tazemetostat without dose interruption, and three recovered during the treatment. The time to recovery for the three patients was 110, 143, and 188 days. Of the remaining three patients, two patients interrupted tazemetostat and one discontinued treatment after 1 cycle of drug interruption due to Grade 2 dysgeusia. One (Patient #14) recovered after approximately 2 weeks of interruption and resumed tazemetostat without dose reduction. The other patient (Patient #10) did not recover despite 1 month of interruption and continued treatment with repeated interruptions for approximately 1 month. The patient (Patient #3) who discontinued treatment developed Grade 1 dysgeusia as of day 107 and Grade 2 dysgeusia as of day 121. Ultimately, treatment was discontinued on day 126 after 1 month of interruption. After the primary analysis, TRAEs of urinary tract infection, peripheral motor neuropathy, and hypogammaglobulinemia were newly reported in one patient each (5%). No newly reported Grade ≥ 3 TRAE occurred. One additional known event of Grade 3 lymphopenia occurred in a total of three patients (15%).

Serious AEs (SAEs) occurred in nine patients (45.0%). Four SAEs (upper respiratory tract inflammation, atypical pneumonia, *Pneumocystis jirovecii* pneumonia, and pneumonia), which were observed in three patients, were considered to be related to tazemetostat. Three patients had reported SAEs of gastric cancer, esophageal carcinoma, periodontitis, or pyrexia after the primary analysis, and three patients developed neoplasm malignancies of gastric cancer, esophageal carcinoma, or non-small cell lung cancer; however, none of those were considered to be related to tazemetostat. No deaths were observed in this study.

**Fig. 5:**
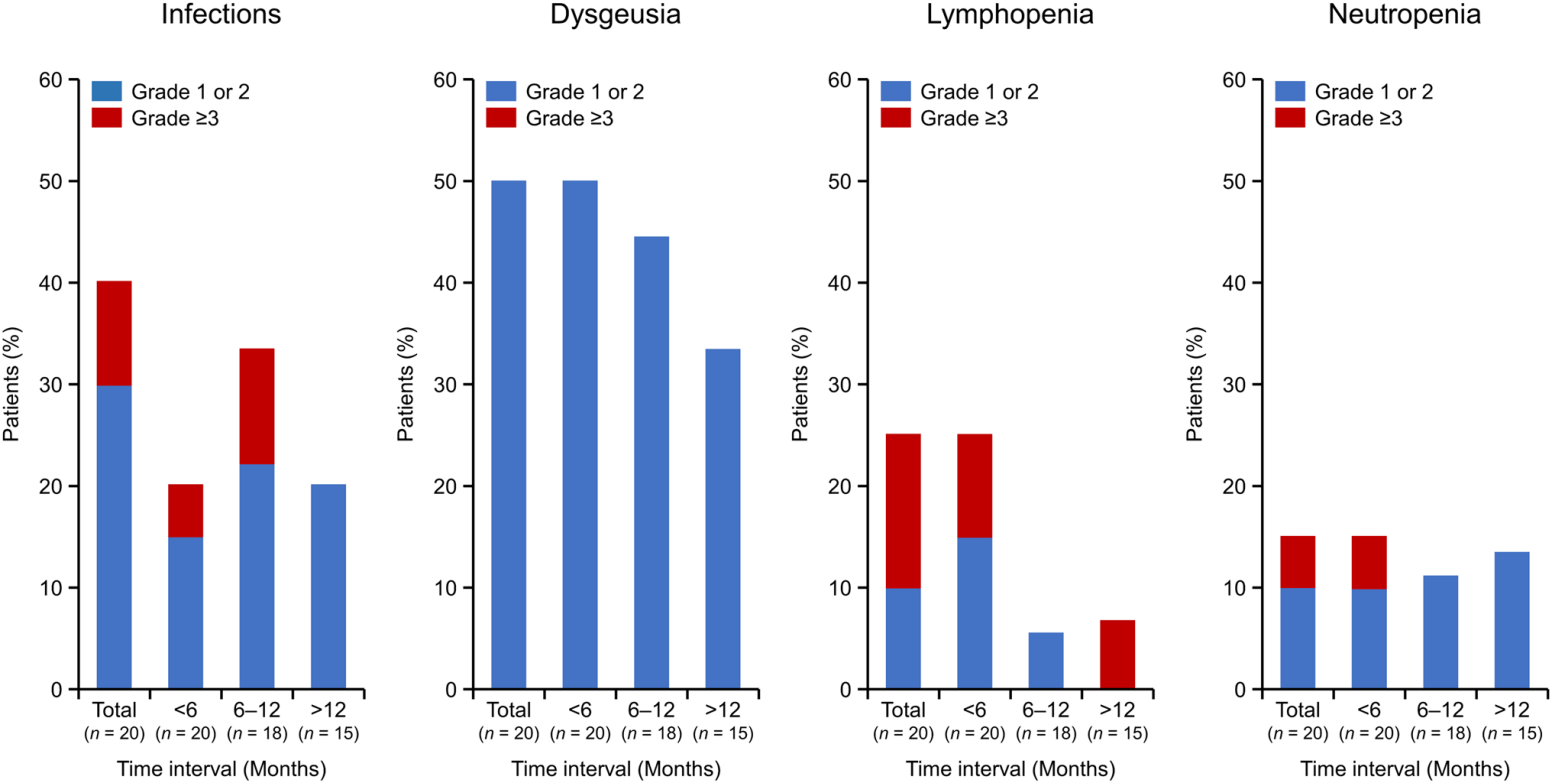
Time to onset of four TRAEs of particular clinical concern (neutropenia, lymphopenia, infections, and dysgeusia). The occurrence of a TRAE during each specific period was counted. If there were multiple occurrences during the same period, the one with the highest grade was selected. Total, *n* = 20; < 6 months, *n* = 20; 6–12 months, *n* = 18; > 12 months, *n* = 15. *TRAE* treatment-related adverse event

To investigate the late-onset toxicity associated with long-term tazemetostat administration, we analyzed the initial timing of dysgeusia as the most frequently reported event, as well as toxicities of infections, lymphopenia, and neutropenia as important identified risks of tazemetostat per the Risk Management Plan of the sponsor (Fig. 5). There were TRAEs classified as infections (MedDRA System Organ Class) in eight patients (40.0%), mostly occurring within 6–12 months (Grade 1–2: 22.2%, Grade 3: 11.1%) from the initial dose of tazemetostat. Grade 3 TRAEs of infection were observed in two patients (*Pneumocystis jirovecii* pneumonia and pneumonia in one patient and atypical pneumonia in one patient), and both patients recovered with dose interruption or discontinuation, respectively. The proportion of patients who experienced dysgeusia was 50.0% (10 of 20 patients) in the early phase of treatment at < 6 months, 44.4% (8 of 18 patients) at 6–12 months, and 33.3% (5/15 patients) in the late phase of treatment at > 12 months. For lymphopenia, which occurred in five patients (25.0%), the first onset of symptoms in all patients occurred in the early phase of treatment at < 6 months (Grade 1–2: 10.0%, Grade 3: 15.0%). For patients who continued treatment, the incidence rate decreased, but one patient (6.7%) developed a Grade 3 AE in the late phase of treatment at > 12 months. For neutropenia, which occurred in three patients (15.0%), the first onset of symptoms in all patients occurred in the early phase of treatment at < 6 months (Grade 1–2: 10.0%, Grade 3: 5.0%). The incidence of neutropenia was generally consistent, ranging from 11.1% to 15.0%, regardless of the length of the treatment period.

## Discussion

This report describes the long-term follow-up of a phase II study of tazemetostat in Japanese patients with B-NHL. With a median follow-up of 35.0 months, the ORR was 70.6%, and the median PFS was not reached per investigator assessment in patients with FL harboring the *EZH2* mutation. Moreover, there were no new safety signals during the long-term follow-up. This safety profile might have enabled long-term continuous treatment with tazemetostat.

With respect to efficacy, the 24-month DOR rate was 90%, and the 24- and 36-month PFS rates were 72.1% and 64.1%, respectively. Moreover, the median number of dosing cycles was 33.0, and the median duration of treatment was 30.2 months. Thus, tazemetostat maintained a durable response with long-term dosing.

Concerning safety, the incidence of TEAEs did not increase remarkably during the follow-up period, and there were no newly reported Grade ≥ 3 TRAEs after the primary analysis [4]. Additionally, tazemetostat was associated with a relatively low incidence of hematologic toxicity throughout the long-term follow-up period compared with other regimens for relapsed/refractory FL [7, 8]. In contrast, it has been associated with a higher incidence of dysgeusia. However, this long-term analysis revealed that dysgeusia did not commonly lead to dose reduction or treatment discontinuation. Furthermore, the first onset of dysgeusia occurred within 6 months of treatment, and there was no increase in its onset after that period. In preclinical studies of tazemetostat, a possible association between tazemetostat and T-lymphoblastic lymphoma was indicated [9]. Whereas one patient with acute myeloid leukemia was reported in the global phase II study of tazemetostat [3], none of the patients in this study developed acute leukemia or lymphoblastic lymphoma during the follow-up period.

This study had several limitations. First, there was a limited number of patients and no control group. Second, the follow-up period was not pre-specified in the protocol, and the response assessment after the primary analysis was only performed by investigators and was not reviewed independently. Furthermore, it should be noted that new treatment options, such as chimeric antigen receptor-T cell therapy (CAR-T) and bispecific antibody therapies, have recently become available for clinical use; thus, the treatment landscape for relapsed/refractory FL is rapidly changing, which makes it difficult to determine the position of tazemetostat from the results of this long-term follow-up study.

In conclusion, we showed the long-term efficacy and safety of tazemetostat for relapsed/refractory FL harboring the *EZH2* mutation. The toxicities of tazemetostat were generally manageable, which may allow for long-term continuous treatment and provide a durable response. These results support the use of tazemetostat as a third-line treatment for relapsed/refractory FL. Currently, a phase III clinical trial (SYMPHONY-1 study, NCT04224493) is ongoing to compare the efficacy and safety of tazemetostat in combination with rituximab and lenalidomide versus the combination of rituximab and lenalidomide for relapsed/refractory FL as second-line treatment.

## Data Availability

The datasets generated during and/or analyzed during the current study are not publicly available but are available from the corresponding author on reasonable request.
